# Characterization of HIV-1 genetic diversity and antiretroviral resistance in the state of Maranhão, Northeast Brazil

**DOI:** 10.1371/journal.pone.0230878

**Published:** 2020-03-27

**Authors:** Élcio Leal, Claudia Regina Arrais, Marta Barreiros, Jessyca Kalynne Farias Rodrigues, Nilviane Pires Silva Sousa, Daniel Duarte Costa, Francisco Dimitre Rodrigo Pereira Santos, Antonio Dantas Silva, Antonia Iracilda e Silva Viana, Allan Kardec Barros, Kledoaldo Lima

**Affiliations:** 1 Institute of Biological Sciences, Federal University of Pará, Belém, PA, Brazil; 2 Graduate Program of the Northeast Biotechnology Network of the Federal University of Maranhão, São Luis, MA, Brazil; 3 Coordination of the Medical Course of the Federal University of Maranhão, Imperatriz, MA, Brazil; 4 Department of Electrical Engineering, Federal University of Maranhão, São Luís, MA, Brazil; 5 Department of Genetics, Federal University of Pernambuco, Recife, PE, Brazil; 6 Food Engineering Coordination, Federal University of Maranhão, Imperatriz, MA, Brazil; 7 Higher Education Unit of Southern Maranhão, Imperatriz, MA, Brazil; 8 Unimed of Empress, Imperatriz, MA, Brazil; 9 Clinical Hospital, Federal University of Pernambuco, Recife, PE, Brazil; 10 European Virus Bioinformatics Center, Jena, Germany; Consejo Superior de Investigaciones Cientificas, SPAIN

## Abstract

The HIV-1 epidemic in Brazil has been growing in northeast and north regions, particularly an increase in AIDS cases among the younger male population has been observed. This study aims to characterize the HIV-1 genetic diversity and to evaluate its antiretroviral resistance profile among individuals presenting virological failure in the state of Maranhão—Brazil. HIV-1 *pol* gene sequences from 633 patients on antiretroviral therapy were obtained from the Department of Surveillance, Prevention and Control of Sexually Transmitted Infections, HIV/AIDS and Viral Hepatitis of the Brazilian Ministry of Health. Phylogenetic and recombination analyses were performed to characterize viral genetic diversity. The presence of antiretroviral resistance mutations was assessed using the HIV Drug Resistance Database online platform of Stanford University. A predominance of subtype B (84.5%) was observed, followed by recombinant BF (9.5%), where more than half of the sequences were dispersed in 3 clusters. Antiretroviral resistance was detected in 74.1% of the sequences, and it was significantly higher for nucleoside analogue reverse-transcriptase inhibitors (NRTIs) than for non-nucleoside analogue reverse-transcriptase inhibitors (NNRTIs) and protease inhibitors (PIs). Inference of putative transmissions clusters identified 11 clusters with 22 query sequences (22/633, 3.5%). Thus, we conclude that continuous monitoring of the molecular epidemiology of HIV-1 is essential for prevention strategies, epidemic control, and treatment adequacy.

## 1 Introduction

Initially, the HIV epidemic in Brazil was reported as the number of AIDS cases per 100,000 inhabitants. However, in recent years, Brazil’s Ministry of Health has also started reporting cases of HIV infection through the Notification Disease Information System. AIDS cases in the country have been stable over the past decade, with a rate of 18.3 cases per 100,000 individuals in 2018, while the number of reported HIV infection rose sharply from seven to 42,000 cases between 2007 and 2017, mainly due to the improvement of information systems [1].

The main HIV-1 subtypes found in Brazil are B, F, C, and BF and BC recombinants [2; 3; 4; 5; 6]. However, Brazil is a country of continental dimensions, with economic, social, and cultural differences, which may interfere with the dynamics of the epidemics. Due to the large territorial proportion, the HIV-1 genetic diversity in Brazil has regional characteristics with subtype B being the most spread in the country [7]. However, some regions have a high proportion of other subtypes and/or recombinants. Some regions of Northeast Brazil have a high proportion of subtype F (~30%) [5] and recombinant BF (18%) [3], as well as circulation of recently identified recombinant circulating forms (CRFs) such as the CRF70 and 71BF [8]. In the southern region, HIV-1 subtype C and its recombinant forms, mainly CRF31_BC, are predominant [9]. In the North and Central-west region, there is a higher prevalence of subtypes B (74–90%) and F (6.7–14%) [10; 11]. Recently, in the Midwest, 2 new CRFs were identified, CRF 90_BF [12] and CRF_99BF [13].

Given the enormous HIV-1 diversity in Brazil, a better knowledge of its genetic variability is crucial for the understanding of inter-subtype recombination mechanisms, vaccine development, viral pathogenesis, transmission pathways, evaluation of antiretroviral drug resistance, and differentiation of therapeutic regimens [14; 15]. Despite all the efforts to combat it, the HIV-1 epidemic has been growing in some regions of Brazil, mainly in the North and Northeast. Besides, there has been an increase in the number of AIDS cases among the younger male population in Brazil, which coincides with the increase in cases among men who have sex with men (from 21.2 to 32.8 per 100,000 inhabitants, from 2008 to 2018) [1]. Thus, understanding HIV-1 molecular epidemiology is essential for prevention and control strategies. This study aims to genetically characterize HIV-1 subtypes and recombinants in the state of Maranhão, northeast Brazil, and to determine their antiretroviral resistance profiles.

## 2 Materials and methods

### 2.1 Study population

HIV-1 *pol* sequences from 633 patients were obtained from the database of the Department of Surveillance, Prevention and Control of Sexually Transmitted Infections, HIV/AIDS and Viral Hepatitis of the Brazilian Ministry of Health (public health service). The HIV-1 *pol* sequences were from individuals undergoing antiretroviral therapy (ART) during the period from 2008 to 2017. The study was approved by the Research Ethics Committee of the Federal University of Maranhão under protocol 3,023,475. All data were fully anonymized. HIV sequences available in a database of the Ministry of Health of Brazil were obtained according to criteria for the evaluation of antiretroviral resistance, with the patient's permission, and the ethics committee waived the requirements for informed consent.

### 2.2 HIV-1 polymerase Gene sequencing (*pol*)

Blood samples were collected in tubes with EDTA anticoagulant as the protocol of the Department of Surveillance, Prevention and Control of Sexually Transmitted Infections, HIV/AIDS and Viral Hepatitis of the Brazilian Ministry of Health for investigation of virological failure in patients undergoing antiretroviral therapy in Brazil. Viral RNA was extracted from plasma using QIAamp Viral mini Kit (Qiagen, Hilden, Germany). The protease (PR) and part of the reverse transcriptase (RT) sequencing of the HIV-1 polymerase gene (*pol*) was performed using the ViroSeqTM HIV-1 Genotyping System (Abbott Laboratories, US) and TRUGENE^®^ HIV-1 Genotyping Assay (Siemens Diagnostics, US) and analyzed using the ABI PRISM 3100 automatic DNA sequencer (Applied Biosystems, US) and OpenGene^®^ Sequencing System (Siemens Diagnostics, US), respectively. Genbank accession numbers: MN971800-MN972432.

### 2.3 Phylogenetic analysis

HIV-1 *pol* sequences were first analyzed using REGA HIV-1 Subtyping Tool–Version 3.0 (dbpartness.stanford.edu:8080/RegaSubtyping/stanford-hiv/typingtool/) for preliminary classification. Reference sequences were obtained from the Los Alamos National Laboratory HIV sequence database (http://www.hiv.lanl.gov/components/sequence/HIV/search/search.html). Alignments, composed by query and reference sequences, were built using the CLUSTAL method implemented in the AliView software [16], followed by manual editing through BioEdit software [17]. The nucleotide substitution models for each alignment were inferred by adopting the maximum likelihood (ML) statistical approach with the Bayesian information criterion implemented the MEGA 7 software [18]. The general time-reversible plus gamma correction (G) and the proportion of invariant sites (I) (GTR + G + I) was the main evolutionary model selected for all alignments. Phylogenetic inferences were performed using maximum likelihood (ML) methods (PhyML) using Seaview version4 [19]. Support branches’ values were computed with the approximate likelihood-ratio test (aLRT) based on the Shimodaira-Hasegawa-like test (SH-test) with Nearest neighbor interchange (NNI) algorithm selected for the tree search. Topologies of the phylogenetic trees were compared with those generated by the Neighbor-Joining (NJ) method under Kimura 2-parameter model with tree topology statistical support evaluated by 1000 replicates (bootstrap) using MEGA 7 software. The phylogenetic trees were visualized with Figtree version 1.4.3 (www.tree.bio.ed.ac.uk/software/figtree/). Reference sequences used for phylogenetic analysis are described in S1 Material.

### 2.4 Antiretroviral resistance analysis

The presence of mutations associated with HIV-1 resistance was determined by submitting the sequences to the HIV Drug Resistance Database online platform at Stanford University (http://hivdb.stanford.edu), which employs the list of major standardized HIV-1 drug resistance mutations (http://hivdb.stanford.edu/assets/media/resistance-mutation-handout-feb2019.b0204a57.pdf).

### 2.5 Recombination analysis

Characterization of the recombinants was performed by SIMPLOT software version 3.5.1 [20]. The following parameters were adopted for the analyses: F84 nucleotide substitution model with 1000 bootstrap support, 140 bp sliding window and 20 bp step size, and empirically determined transition/transverse ratio for each alignment. Gaps were automatically removed from the alignment. Sequences were considered recombinant when they had a bootstrap of 70% for more than one HIV-1 subtype along the nucleotide sequence.

### 2.6 Transmission cluster inference

Transmissions Clusters networks were inferred using HIV-TRACE online tool (Transmission Cluster Engine) [21] (hivtrace.datamonkey.org/hivtrace). Each query sequence alignment for subtypes B, F and BF recombinants were analyzed separately. Firstly, all sequences were aligned to HXB2 reference sequence. Putative network transmission between query sequences was considered if the pairwise distance computed were ≤0.020 substitutions/site (distance threshold) measure by TN93 substitution model, because this threshold was previously tested in HIV-1 epidemiologically related clusters [22]. An ambiguity fraction of 0.05 was adopted, that is only sequences with <5% ambiguities were retained [21]. HIV-TRACE analysis has been made with the exclusion of drug resistance-associated mutations, due to the possible effect of convergent evolution caused by antiretroviral resistance pressure that has the potential to confound evolutionary analysis [23].

### 2.7 Statistical analysis

The Person chi-squared and Fisher’s exact tests were used for comparison of secondary resistance frequencies among antiretroviral drugs. Data were analyzed using EpiInfo^TM^ version 7.2.2.16.

## 3 Results

### 3.1 HIV-1 genetic diversity

**Fig 1:**
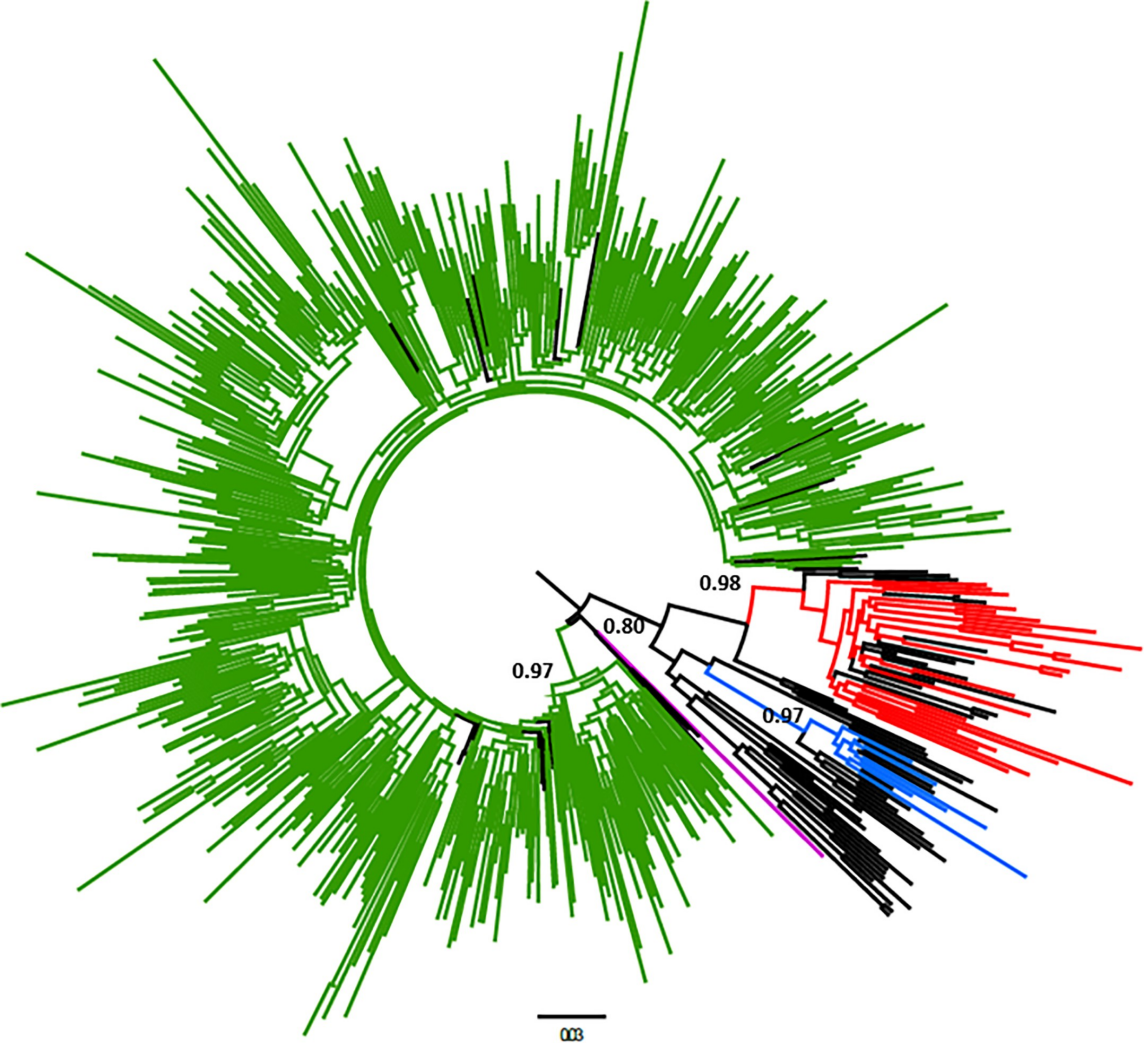
Phylogenetic tree of HIV-1 pol sequences obtained from Northeast—Brazil. The maximum-likelihood tree obtained with 1038 bp corresponding to the protease region and part of the reverse transcriptase. A total of 535 HIV-1 subtype B query sequences branched (aLTR = 0.97) with 22 HIV-1 B reference sequences (depicted in green). Twenty-five HIV-1 subtype F query sequences branched (aLTR = 0.98) with 17 references sequences (red color). Six HIV-1 subtype C query sequences were identified (aLTR = 0.97) (blue color). Query sequence 17BR_MA6262 clustered with HIV-1 subtype D references sequences (aLTR = 0.80) (purple color). Reference sequences of HIV-1 subtypes A1, A2, A3, A4, A6, B, C, D, F1, F2, G, H, J, and K were used to phylogenetic analysis, described in the supplementary material (black color).

Among HIV-1 *pol* sequences, subtype B prevails (n = 535, 84.5%; 95%CI: 81.7–87.3%), followed by a high frequency of BF recombinants (n = 60, 9.5%; 95%CI: 7.4–12%) and lower proportions of subtypes F (n = 25, 3.9%; 95%CI: 2.7–5.8%), C (n = 06, 0.9%; 95%CI: 0.4–2.1%), and BC recombinants (n = 06, 0.9%; 95%CI: 0.4–2.1%). One sequence was identified as HIV-1 subtype D (17BR_MA6262), obtained by a female patient in 2017. 57.2% of individuals were male (n = 362), while women comprised 42.8% (n = 271). Fig 1 represents the phylogenetic analysis of sequences belonging to subtypes B, F and C.

**Fig 2:**
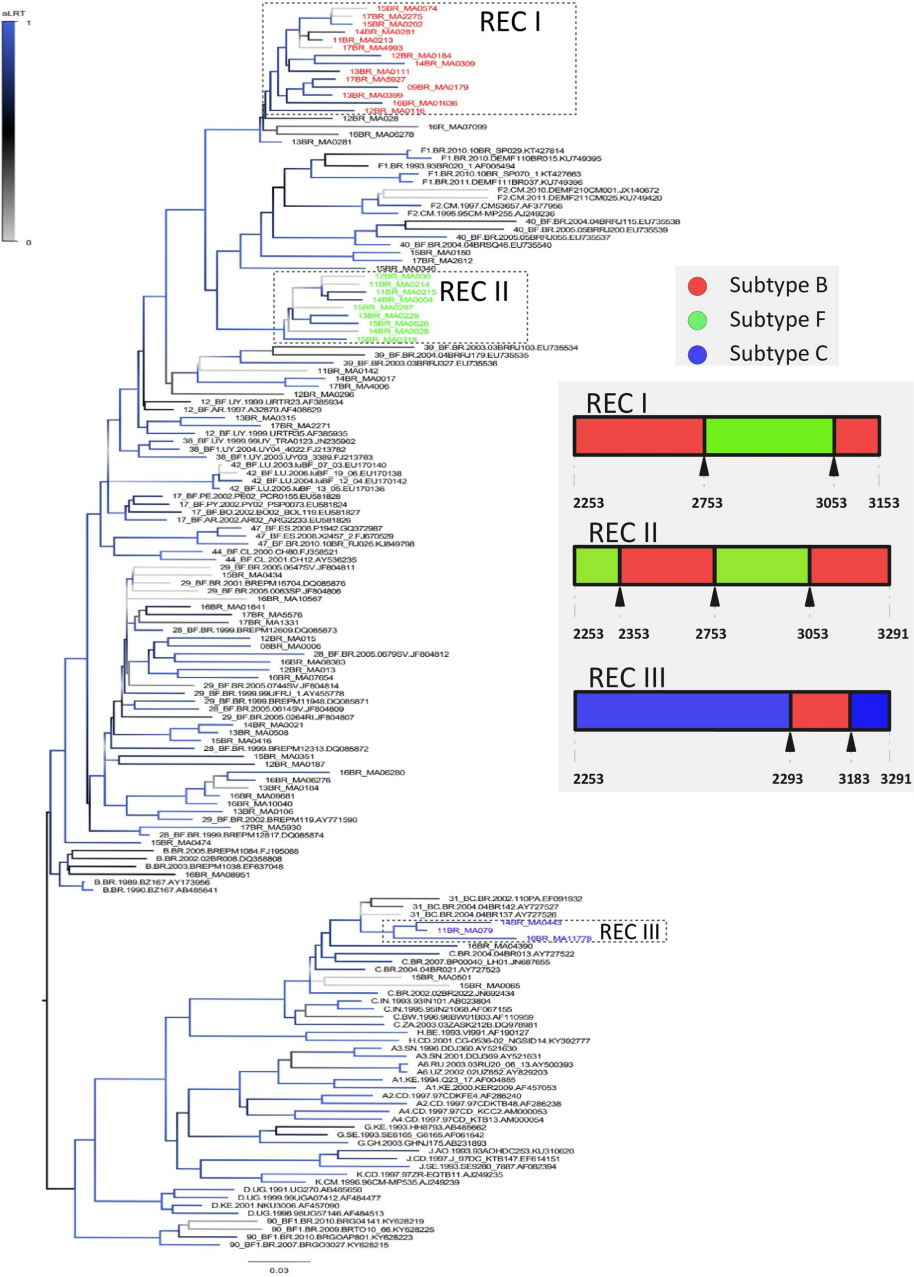
Phylogenetic analysis of HIV-1 BF and BC recombinants inferred with 1038 bp corresponding to the protease region and part of the reverse transcriptase, obtained in the state of Maranhão (Northeast Brazil). Phylogenetic inference included reference sequences of various subtypes and the major BF and BC recombinants found in Brazil. Clusters of recombinant sequences were assigned by colors: green (9 sequences), red (14 sequences) and blue (three sequences). The mosaic pattern of BF and BC is shown in the figure (gray area). The diagram colors indicate the genome regions of protease composed by distinct parental subtypes and the arrows indicate the location (numbers correspond to the nucleotide position of the reference HIV-1 strain HXB2) of breakpoints. More detailed features of recombinant strains of this study were included in the S1 Material.

Despite the low frequency of subtype C, an equal proportion between C and BC recombinants has been observed. Out of 6 BC recombinants detected 4 had a CRF31-like pattern grouped into a cluster related to CRF31_BC and 2 other strains have shown distinct recombination patterns (Fig 2 and S1 Fig–S1 Material). Further, 42.6% of the BF recombinants (25/60) were divided into 2 clusters (Fig 2). Those clusters have not shared recombination breakpoints with other CRFs BF (see breakpoints in the caption in Fig 2). BF recombinant cluster with nine sequences (shown in green in Fig 2) have breakpoints, in the analyzed genomic region, closer to CRFs 39 and 90BF, however, none evolutionary relationship among them in phylogenetic analysis has been seen. The largest BF cluster with fourteen sequences (colored in red in Fig 2), has two breakpoints and the other BF cluster (nine sequences) are composed of recombinant strains with three breakpoints. The diagram in Fig 2 shows the parental composition and locations of breakpoints in the protease of recombinant strains identified in our study. The recombination patterns of these two clusters are shown in S2 Fig. Twenty-one query sequences showed phylogenetic relationships with CRFs 28e29_BF. Among them, two clusters were identified, one with three (aLTR: 1.00) and another one with five query sequences (aLTR: 1.00). Nevertheless, none sequence revealed a recombination pattern similar to the described CRFs (S3 Fig–S1 Material).

### 3.2 Antiretroviral resistance

**Fig 3:**
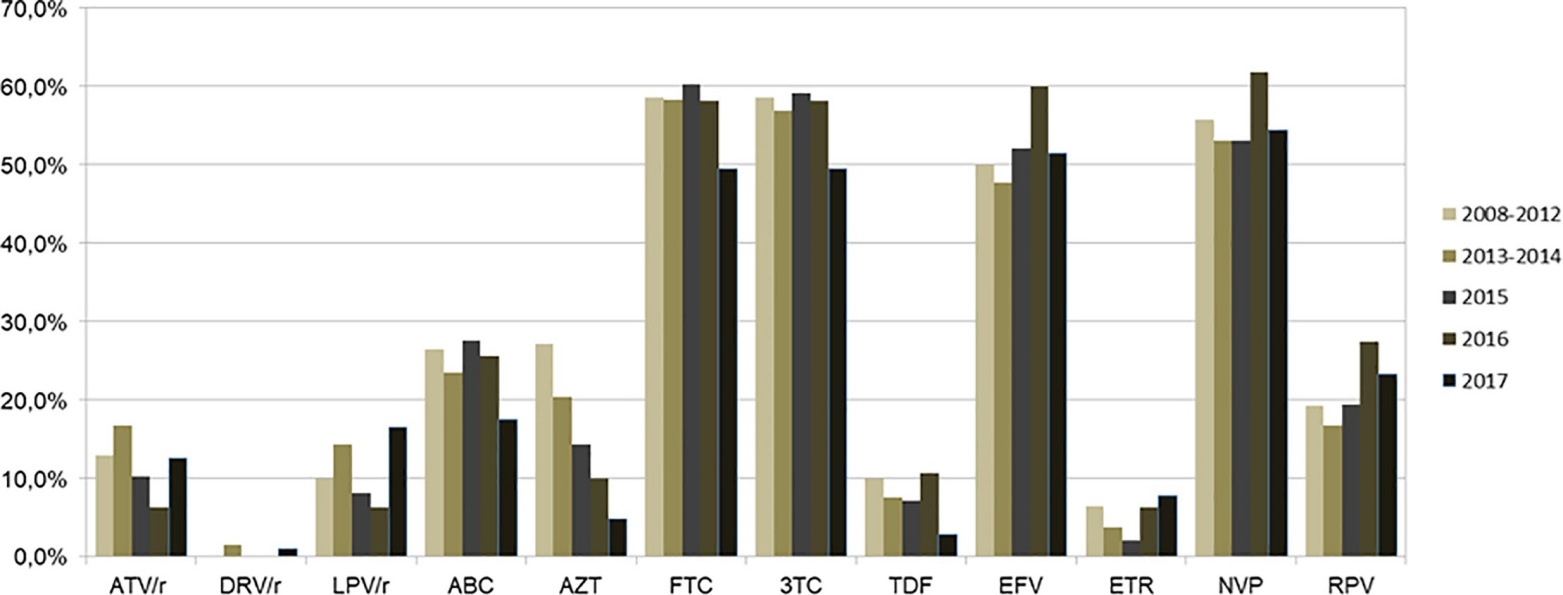
Resistance of HIV-1 to antiretroviral drugs. The evaluated samples were collected in the period between 2008 and 2017 in the state of Maranhão, Brazil. In the period 2008–2012, data of 2010 were excluded. ATV/r: Atazavanir/ritonavir, DRV/r: Darunavir/ritonavir, LPV/r: Lopinavir/ritonavir, ABC: Abacavir, AZT: Zidovudine, FTC: Emtricitabine, 3TC: Lamivudine, TDF: Tenofovir, EFV: Efavirenz, ETR: Etravirine, NVP: Nevirapine, RPV: Rilpivirine.

Antiretroviral resistance analysis was performed for PI, NRTIs and NNRTIs. Seventy-four percent (95%CI: 70.5–77.4) of the analyzed sequences displayed resistance to at least one of the tested inhibitors. It was observed that the frequency of resistance to NRTIs (65.2%, 95% CI: 61.5–68.9) was significantly higher than the one to NNRTIs (57.8%, 95%CI: 53.9–61.6) and PIs (18.6%, 95%CI: 15.8–21.9) (p <0.001), and the frequency of resistance for NNRTIs was higher than for PIs (p <0.001). When analyzing antiretroviral resistance in relation to different times, no significant difference in temporal variations was found (Table 1). The main resistance mutations that we identified were D30N, M46I, I54LV, V82A, N88D, and L90M for PIs; M41L, K65R, D67N, K70R, M184V, L210W, and T215FY for NRTIs; and L100I, K103N, and G190A for NNRTIs. Among PIs, Darunavir/ritonavir had very low resistance rates, only 3/633 (0.5%, 95%CI: 0.2–1.4), while atazanavir/ritonavir and lopinavir/ritonavir have shown significantly higher average frequencies of 11.5% (95%CI: 9.3–14.3) and 10.7 (95%CI: 8.6–13.4), respectively (p< 0.0001). Emtricitabine and Lamivudine had higher resistance rates (around 50–60%) than NRTIs (p< 0.0001), while Tenofovir was the NRTI with the lowest secondary resistance level (average rate of 8%, 95%CI: 6.2–10.4, p<0.0001). Among NNRTIs, higher resistances were observed against Efavirenz and Nevirapine than Etravirine and Rilpivirine (p <0.0001). Rilpivirine has been the NNRTI with a lower resistance level (average rate of 5.4%, 95%CI: 3.9–7.4, p< 0.0001) (Fig 3).

**Table 1:** Antiretroviral resistance of individuals in virological failure in the state of Maranhão (Northeast Brazil).

	Resistance Mutation Frequency					
Antiretroviral Drug Types	Total N = 633	2008–2012^1^ (N = 140)	2013–2014 (N = 132)	2015 (N = 98)	2016 (N = 160)	2017 (N = 103)
	n (%) (CI95%)	n (%) (CI95%)	n (%) (CI95%)	n (%) (CI95%)	n (%) (CI95%)	n (%) (CI95%)
PI^2^	118 (18.6) (15.8–21.9)	28 (20) (14.2–27.4)	30 (22.7) (16.4–36.6)	17 (17.3) (11.1–26.0)	25 (15.6) (10.8–22.0)	18 (17.5) (11.3–25.9)
NRTI/NNRTI	463 (73.1) (69.6–76.4)	101 (72.1) (64.2–78.9)	93 (70.5) (62.2–77.6)	71 (72.4 (62.9–80.3)	134 (83.8) (77.3–88.7)	64 (62.1) (52.5–70.9)
NRTI^3^	413 (65.2) (61.5–68.9)	94 (67.1) (59.0–74.4)	83 (62.9) (54.4–70.6)	68 (69.4) (59.7–77.6)	108 (67.5) (59.9–74.3)	57 (55.3) (45.7–64.6)
NNRTI^4^	366 (57.8) (53.9–61.6)	81 (57.9) (49.6–65.7)	71 (53.8) (45.3–62.1)	53 (54.1) (44.2–63.6)	104 (65) (57.3–72.0)	57 (55.3) (45.7–64.6)
Any resistance	469 (74.1) (70.5–77.4)	102 (72.9) (65–79.5)	96 (72.7) (64.6–79.6)	72 (73.5) (64.0–81.2)	135 (84.4) (78.0–89.2)	64 (62.1) (52.5–70.9)

^1^ In this period, 2010 was excluded (missing data)

^2^ PI: Protease Inhibitors

^3^ NRTI: Nucleoside Analog Reverse Transcriptase Inhibitors

^4^ NNRTI: Non-analogous Nucleoside Reverse Transcriptase Inhibitors.

### 3.3 Transmission clusters

Transmission cluster analysis identified 11 clusters containing 22 query sequences (22/633, 3.5%). All putative transmissions clusters contained only two individuals. Most clusters were composed of individuals infected with subtype B (n = 09). Subtype F and BF recombinants were identified in one cluster each. Only five women have been shown in the clusters (05/22) and the query sequences belonging to the transmissions clusters have been isolated among the sampling period 2014–2017 (Table 2).

**Table 2:** Characteristics of transmissions clusters identified in northeastern Brazil.

Cluster No	Sequences	Gender	Sampling Period	Subtype
1	17BR_MA0423	Male	2017	F
	17BR_MA2934	Female	2017	F
2	15BR_MA0202	Female	2015	BF
	17BR_MA2275	Male	2017	BF
3	14BR_MA0018	Male	2014	B
	14BR_MA0019	Male	2014	B
4	15BR_MA0252	Female	2015	B
	17BR_MA5007	Male	2017	B
5	15BR_MA0155	Male	2015	B
	17BR_MA2610	Female	2017	B
6	16BR_MA09679	Male	2016	B
	16BR_MA09682	Female	2016	B
7	14BR_MA0005	Male	2014	B
	16BR_MA06279	Male	2016	B
8	16BR_MA07108	Male	2016	B
	17BR_MA4991	Male	2017	B
9	15BR_MA0247	Male	2015	B
	17BR_MA1998	Male	2017	B
10	15BR_MA0152	Male	2015	B
	15BR_MA0153	Male	2015	B
11	16BR_MA07350	Male	2016	B
	16BR_MA11263	Male	2016	B

## 4 Discussion

A High frequency of subtype B (84.7%) was detected, and among non-B subtypes, BF recombinants (9.5%) were highlighted. A high level of antiretroviral resistance was observed, with 74.1% (95%CI: 70.5–77.4) of the strains presenting resistance to at least one of the analyzed classes of drugs (NRTIs, NNRTIs, and PIs). However, the frequency of resistance to PIs was significantly lower than the resistance to NRTIs and NNRTIs (p <0.001), with 18.6% (95%CI: 15.8–21.9), 65.2% (95%CI: 61.5–68.9) and 57.8% (95%CI: 53.9–61.6), respectively. The high proportion of HIV-1 subtype B detected in our study corroborates with previous phylogenetic data on HIV-1 genetic diversity in Maranhão state, which also showed a high rate of HIV-1 B (81.1%) [24] and in neighboring states, such as Piauí (HIV-1 B = 86.5%) and Pará (HIV-1 B = 90%) [25]. However, as Brazil is a country of continental dimensions, there is variation in the HIV-1 genetic diversity in the same macroregion. Thus, in the Northeast, there are states with lower proportions of HIV-1 B, as Pernambuco, where the subtype is F common (31.4%) [5], and Bahia with a frequency of 18.6% for BF recombinants [3]. BF recombinants were detected in 9.5% of all sequences in our study. This may be related to the fact that the state of Maranhão is closely located to places with a large circulation of these recombinants [26], including areas where new CRFs BF were identified, such as CRF90_BF in Tocantins and Goiás and CRF99_BF in Goiás [12; 13]. It is important to highlight the cluster of recombinant like-31BC, whose presence is very common in the southern region of the country. Another interesting point is the large number of query sequences (n = 21, 35% of BF recombinants) phylogenetically related to recombinants 28 and 29BF. This can be partially explained by the high flux of tourists that came from Southeastern Brazil which is the place of origin of recombinants 28 and 29BF.

Antiretroviral resistance was evaluated in patients referred for HIV-1 genotyping due to virological failure, thus all analyzed patients have been undergoing treatment. This explains the high rate of secondary resistance (74.1%, 70.5–77.4%). PIs showed a lower proportion of resistance when compared to NRTIs and NNRTIs. Moreover, Darunavir/ritonavir resistance was very low, as only 3 strains showed resistance to it, which reveals its excellent applicability as rescue therapy. To our knowledge, this is the first study with a large number of individuals evaluating the HIV-1 antiretroviral resistance and diversity in patients who had started antiretroviral therapy in the state of Maranhão, one of the places with the lowest Human Development Index in Brazil. Another research, carried out in the same state by [24], was performed on samples from naive patients and identified a frequency of 3.8% (95% CI: 1.2–8.9) of primary resistance mutations. Only mutations for NRTIs and NNRTIs resistance were detected, demonstrating the low circulation of primary mutations to PIs in untreated patients. On the other hand, a study evaluating patients with therapeutic failure in large cities in Brazil, including the Northeast region, also indicated a higher level of resistance to NRTIs and NNRTIs, but with a lower frequency of mutations for PIs resistance in both naive and treated individuals [27]. Among NNRTIs, higher resistances were observed against Efavirenz and Nevirapine, which was largely used NNRTIs in Brazil as the recommended first-line regimen, being replaced, in 2017, by the integrase inhibitor dolutegravir. Although Tenofovir is recommended as the first-line regimen, it has been presented the lowest rate of secondary antiretroviral resistance among NRTIs, which gives it a great treatment option.

In 2017, the Brazilian Ministry of Health began offering pre-exposure prophylaxis (PrEP), which is still expanding. PrEP is part of the preventive administration of Tenofovir and Entricitabine in specific groups of HIV-1 positive individuals [28]. Our data showed a much lower resistance to Tenofovir than Entricitabine, which may assure the effectiveness of PrEP, but a continuous assessment of the HIV-1 infected population for antiretroviral resistance will be required in view of the adoption of this new prophylactic measure. One of the limitations of this study is the lack of assessment of resistance to Integrase Inhibitors. However, this class of antiretroviral was included in the treatment of newly diagnosed individuals for HIV-1 only in 2017, just in the final period of our research.

Individuals in virological failure do not reflect the current scenery of the HIV-1 epidemic, as these individuals were usually diagnosed and have already started antiretroviral therapy and thereby they are not in the recent infection phase. Thus, to have a more up-to-date parameter on the HIV-1 genetic diversity, viral sequencing of newly infected individuals should be performed. Moreover, it is observed that antiretroviral therapy acts on the natural selection of resistant strains, which may influence the evolutionary and phylogenetic analyses.

We identified 11 transmission clusters containing 22 query sequences. Most of the patients have been men (17/22), harboring subtype B (18/22) and had their sampling period among 2014–2017. In HIV-1 subtype B, two-thirds of the clusters networks were composed of male patients (6/9), with no information about of sexual exposure. Another study in Northeast Brazil [29] showed a higher number of men who have sex with men infected with HIV-1 subtype B when compared with non-B subtypes, nevertheless without statistical significance (p = 0.06).

The present study indicates a predominance of subtype B and BF recombinants. One of the important factors in determining the HIV-1 genetic diversity is the identification of possible strains that are more likely to result in disease progression. Some studies in Brazil have linked BF recombinants with faster progression to AIDS-defining events [30] or faster CD4+ lymphocyte count decline [31]. Thus, the high frequency of these recombinants may be related to a greater chance of disease progression. In addition, BF recombinants showed a higher frequency of PIs resistance, although with borderline statistical significance (data not shown). The presented data reveal the importance of investigating the molecular epidemiology of HIV-1, as this will enable us to monitor the spread or retraction of the epidemic and of antiretroviral resistance. In addition, molecular analyses can provide important data for constant update and optimization of therapy, implementation of preventive measures, and evaluation of transmission of the various HIV-1 subtypes and recombinants.

## 5 Conclusions

The study was conducted in patients with antiretroviral virological failure from Northeast Brazil. High HIV-1 genetic diversity was observed, with a predominance of subtype B (84.7%) and BF recombinants (9.5%). A high frequency of secondary antiretroviral resistance mutations (74.1%) was also demonstrated, with a higher rate in Nucleoside and Non-nucleoside Reverse Transcriptase Inhibitors than in Protease Inhibitors. Emtricitabine, lamivudine, efavirenz, and nevirapine were the antiretroviral drugs with the most resistance.

## Supporting information

S1 Material (PDF)
