# Supplementary material for: Characterization of HIV-1 genetic diversity and antiretroviral resistance in the state of Maranhão, Northeast Brazil
Source: PLoS One. 2020 Mar 27;15(3):e0230878. doi: 10.1371/journal.pone.0230878 (PMC7100926; doi:10.1371/journal.pone.0230878)
Supplement: S1 Material — (PDF) [file pone.0230878.s001.pdf]

# Characterization of HIV-1 genetic diversity and antiretroviral resistance in the state of Maranhão, Northeast Brazil

Élcio Leal[a*], Claudia Regina Arrais Rosa[b,c], Marta Barreiros[d], Jessyca Kalynne Farias Rodrigues[e], Nilviane Pires S. Sousa[b,d], Daniel Duarte Costa[f], Francisco Dimitre Rodrigo Pereira Santos[g], Antonio Dantas Silva[h], Antonia Iracilda e Silva Viana[c], Allan Kardec Barros[b,d], Kledoaldo Lima[i,j]

[a] Institute of Biological Sciences, Federal University of Pará, Belém, PA, Brazil

[b] Graduate Program of the Northeast Biotechnology Network of the Federal University of Maranhão, São Luis, MA, Brazil

[c] Coordination of the Medical Course of the Federal University of Maranhão, Imperatriz, MA, Brazil

[d] Department of Electrical Engineering, Federal University of Maranhão, São Luís, MA, Brazil

[e] Department of Genetics, Federal University of Pernambuco, Recife, PE, Brazil

[f] Food Engineering Coordination, Federal University of Maranhão, Imperatriz, MA, Brazil

[g] Higher Education Unit of Southern Maranhão, Imperatriz, MA, Brazil

[h] Unimed of Empress, Imperatriz, MA, Brazil

[i] Clinical Hospital, Federal University of Pernambuco, Recife, PE, Brazil

[j] European Virus Bioinformatics Center, Leutragraben 1, 07743 Jena, Germany

* Corresponding author:

**Email: elcioleal@gmail.com (EL)**

**SUPPLEMENTARY MATERIAL**

**HIV-1 Sequences used in the phylogenetic analysis shown in Figure 1:**

A1.KE.1994.Q23_17.AF004885;A1.KE.2000.KER2009.AF457053;A2.CD.1997.97CDKFE4.AF286240;A2.CD.1997.97CDKTB48.AF286238;A3.SN.1996.DDJ360.AY521630;A3.SN.2001.DDJ369.AY521631; A4.CD.1997.97CD_KCC2.AM000053; A4.CD.1997.97CD_KTB13.AM000054; A6.RU.2003.03RU20_06_13.AY500393; A6.UZ.2002.02UZ652.AY829203; B.AR.1999.ARMA132.AY037282; B.AR.2000.ARMS008.AY037269; B.AR.2002.02AR114146.DQ383746; B.AU.1986.MBC200.AF042100; B.BR.1989.BZ167.AY173956; B.BR.1990.BZ167.AB485641; B.BR.2002.02BR002.DQ358805; B.BR.2002.02BR008.DQ358808; B.BR.2003.BREPM1038.EF637048; B.BR.2003.BREPM2012.EF637046; B.BR.2004.BREPM1066.FJ195090; B.BR.2004.BREPM1070.FJ195086; B.BR.2005.BREPM1081.FJ195091; B.BR.2005.BREPM1084.FJ195088; B.BR.2005.BREPM1093.FJ195089; B.FR.1983.HXB2-LAI-IIIB-BRU.K03455; B.FR.1985.NL43xWC001.AF003887; B.US.1985.Ba_L.AB221005; B.US.1986.AD87_ADA.AF004394; B.US.1991.DH12_3.AF069140; B.US.1991.US2.AB485640; B.US.1997.ARES2.AB078005; C.BR.2002.02BR2022.JN692434; C.BR.2004.04BR013.AY727522;; C.BR.2004.04BR021.AY727523; C.BR.2007.BP00040_LH01.JN687655; C.BW.1996.96BW01B03.AF110959; C.IN.1993.93IN101.AB023804; C.IN.1995.95IN21068.AF067155; C.ZA.2003.03ZASK212B.DQ978981;; D.KE.2001.NKU3006.AF457090; D.UG.1991.UG270.AB485650; D.UG.1998.98UG57146.AF484513; D.UG.1999.99UGA07412.AF484477; F1.AR.2002.ARE933.DQ189088; F1.BR.1989.BZ126.AY173957; F1.BR.1990.BZ163.AB485656; F1.BR.1993.93BR020_1.AF005494; F1.BR.2002.02BR082.FJ771006; F1.BR.2007.07BR844.FJ771010; F1.BR.2010.10BR_PE107.KJ849782; F1.BR.2010.10BR_RJ015.KJ849791; F1.BR.2010.10BR_RJ055.KT427774; F1.BR.2010.10BR_SP029.KT427814; F1.BR.2010.10BR_SP070_1.KT427663; F1.BR.2010.DEMF110BR015.KU749395; F1.BR.2011.DEMF111BR037.KU749396; F1.ES.-.P1146.DQ979023; F1.FI.1993.FIN9363.AF075703; F1.FR.1996.96FR-MP411.AJ249238; F1.RO.1996.BCI_R07.AB485658; F2.CM.1995.95CM-MP255.AJ249236; F2.CM.1997.CM53657.AF377956; F2.CM.2010.DEMF210CM001.JX140672; F2.CM.2011.DEMF211CM025.KU749420; G.GH.2003.GHNJ175.AB231893; G.KE.1993.HH8793.AB485662; G.SE.1993.SE6165_G6165.AF061642; H.BE.1993.VI991.AF190127; H.CD.2001.CG-0536-02_NGSID14.KY392777; J.AO.1993.93AOHDC253.KU310620; J.CD.1997.J_97DC_KTB147.EF614151; J.SE.1993.SE9280_7887.AF082394; K.CD.1997.97ZR-EQTB11.AJ249235; K.CM.1996.96CM-MP535.AJ249239.

**SUPPLEMENTARY MATERIAL**

**HIV-1 Sequences used in the phylogenetic analysis shown in Figure 2:**

40_BF.BR.2004.04BRRJ115.EU735538; 40_BF.BR.2005.05BRRJ200.EU735539; 40_BF.BR.2004.04BRSQ46.EU735540; 40_BF.BR.2005.05BRRJ055.EU735537; 90_BF1.BR.2009.BRTO10_66.KY628225; 90_BF1.BR.2010.BRG04141.KY628219; 90_BF1.BR.2007.BRGO3027.KY628215; 90_BF1.BR.2010.BRGOAP801.KY628223; 39_BF.BR.2003.03BRRJ103.EU735534; 39_BF.BR.2003.03BRRJ327.EU735536; F2.CM.2011.DEMF211CM025.KU749420; F2.CM.2010.DEMF210CM001.JX140672; F2.CM.1995.95CM-MP255.AJ249236; F2.CM.1997.CM53657.AF377956; 39_BF.BR.2004.04BRRJ179.EU735535; F1.BR.2010.10BR_SP029.KT427814; F1.BR.2010.DEMF110BR015.KU749395; F1.BR.1993.93BR020_1.AF005494; F1.BR.2010.10BR_SP070_1.KT427663; F1.BR.2011.DEMF111BR037.KU749396; 28_BF.BR.2005.0679SV.JF804812; D.UG.1991.UG270.AB485650; D.UG.1999.99UGA07412.AF484477; D.KE.2001.NKU3006.AF457090; D.UG.1998.98UG57146.AF484513; 47_BF.BR.2010.10BR_RJ026.KJ849798; 47_BF.ES.2008.P1942.GQ372987; 47_BF.ES.2008.X2457_2.FJ670529; 42_BF.LU.2003.luBF_07_03.EU170140; 42_BF.LU.2005.luBF_13_05.EU170136; 42_BF.LU.2004.luBF_12_04.EU170142; 42_BF.LU.2006.luBF_19_06.EU170138; 29_BF.BR.2002.BREPM119.AY771590; B.BR.2005.BREPM1084.FJ195088; B.BR.2002.02BR008.DQ358808; B.BR.1989.BZ167.AY173956; B.BR.1990.BZ167.AB485641; B.BR.2003.BREPM1038.EF637048; 44_BF.CL.2000.CH80.FJ358521; 44_BF.CL.2001.CH12.AY536235; 29_BF.BR.2005.0264RI.JF804807; 17_BF.BO.2002.BO02_BOL119.EU581827; 17_BF.AR.2002.AR02_ARG2233.EU581826; 17_BF.PE.2002.PE02_PCR0155.EU581828; 17_BF.PY.2002.PY02_PSP0073.EU581824; 12_BF.UY.1999.URTR35.AF385935; 12_BF.AR.1997.A32879.AF408629; 12_BF.UY.1999.URTR23.AF385934; 38_BF1.UY.2003.UY03_3389.FJ213783; 38_BF.UY.1999.99UY_TRA0123.JN235962; 38_BF1.UY.2004.UY04_4022.FJ213782; 28_BF.BR.1999.BREPM12313.DQ085872; 29_BF.BR.2005.0647SV.JF804811; 29_BF.BR.2005.0744SV.JF804814; 29_BF.BR.1999.BREPM11948.DQ085871; 29_BF.BR.2005.0063SP.JF804806; 28_BF.BR.2005.0614SV.JF804809; 29_BF.BR.1999.99UFRJ_1.AY455778; 28_BF.BR.1999.BREPM12817.DQ085874; 28_BF.BR.1999.BREPM12609.DQ085873; 29_BF.BR.2001.BREPM16704.DQ085876; H.BE.1993.VI991.AF190127; H.CD.2001.CG; 0536-02_NGSID14.KY392777; C.BR.2004.04BR013.AY727522; C.ZA.2003.03ZASK212B.DQ978981; C.BW.1996.96BW01B03.AF110959; C.IN.1993.93IN101.AB023804; C.IN.1995.95IN21068.AF067155; C.BR.2002.02BR2022.JN692434; 31_BC.BR.2002.110PA.EF091932; 31_BC.BR.2004.04BR137.AY727526; 31_BC.BR.2004.04BR142.AY727527; C.BR.2007.BP00040_LH01.JN687655; C.BR.2004.04BR021.AY727523; J.SE.1993.SE9280_7887.AF082394 J.AO.1993.93AOHDC253.KU310620; J.CD.1997.J_97DC_KTB147.EF614151; A2.CD.1997.97CDKFE4.AF286240; A2.CD.1997.97CDKTB48.AF286238; A4.CD.1997.97CD_KCC2.AM000053; A4.CD.1997.97CD_KTB13.AM000054; A6.RU.2003.03RU20_06_13.AY500393; A6.UZ.2002.02UZ652.AY829203; A3.SN.1996.DDJ360.AY521630; A3.SN.2001.DDJ369.AY521631; A1.KE.1994.Q23_17.AF004885; A1.KE.2000.KER2009.AF457053; K.CD.1997.97ZR-EQTB11.AJ249235; K.CM.1996.96CM; MP535.AJ249239; G.GH.2003.GHNJ175.AB231893; G.KE.1993.HH8793.AB485662; G.SE.1993.SE6165_G6165.AF061642

## SUPPLEMENTARY MATERIAL Figure S1

11BRMA079

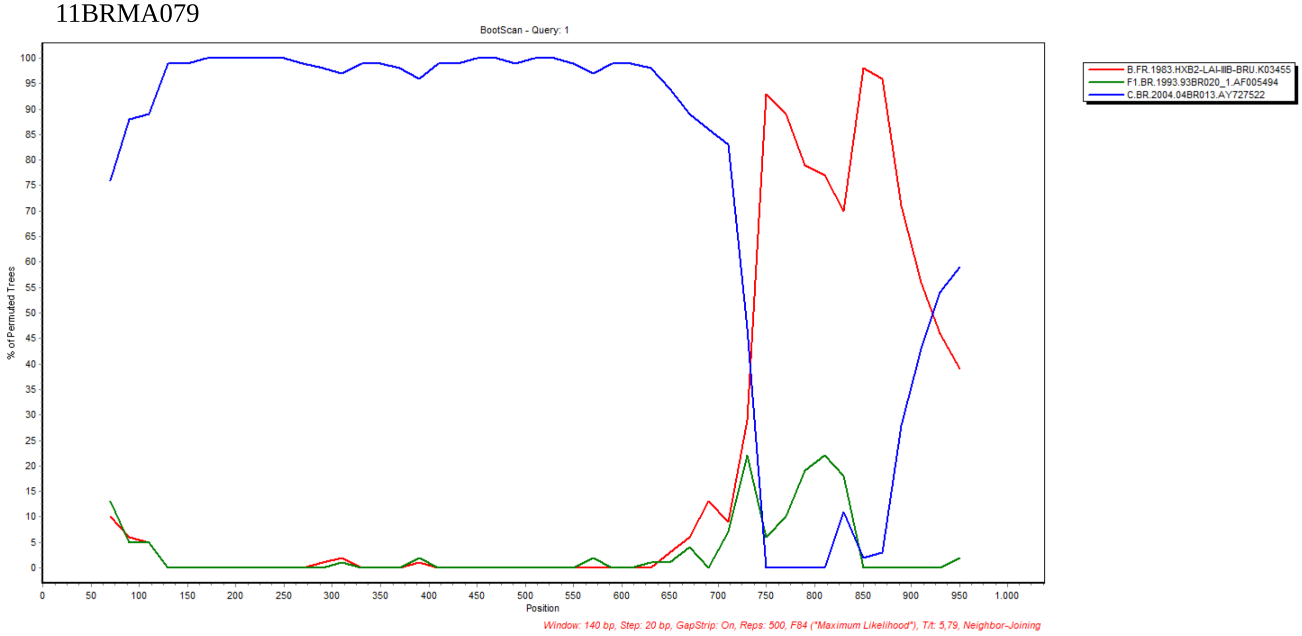


14BRMA0443

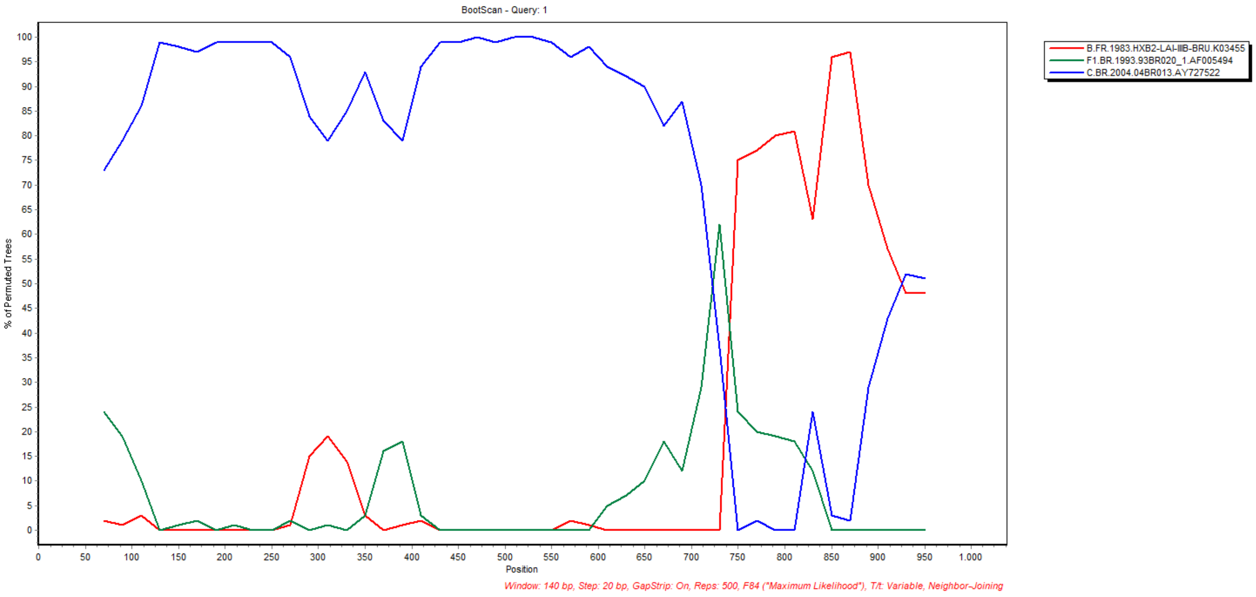

16BRMA11778

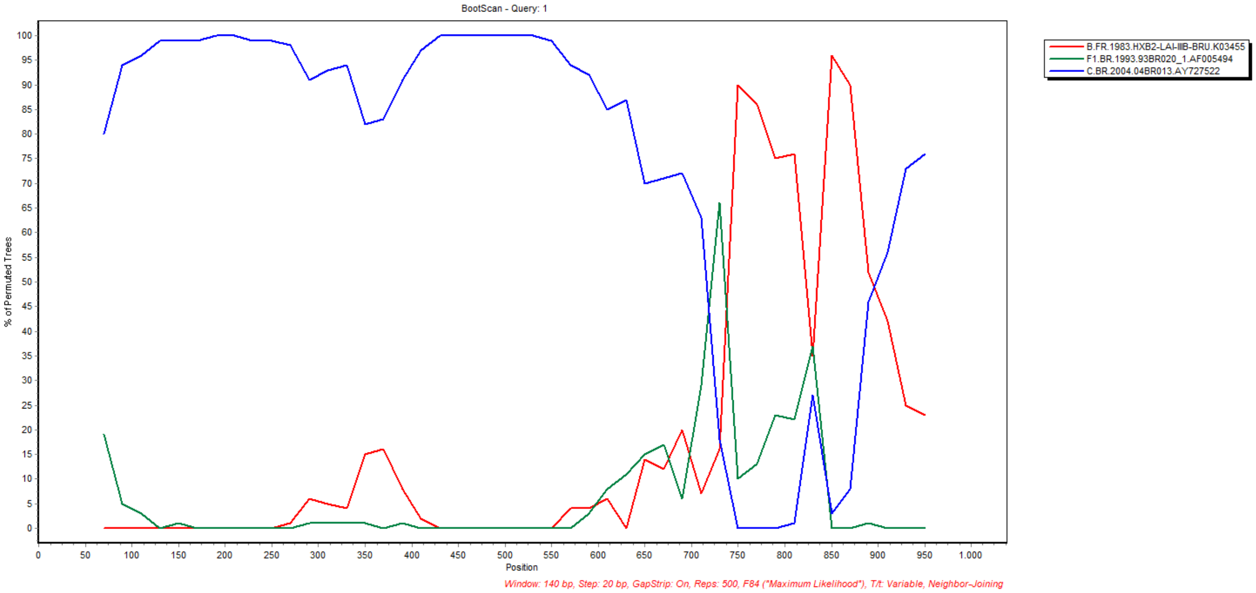


15BRMA0065

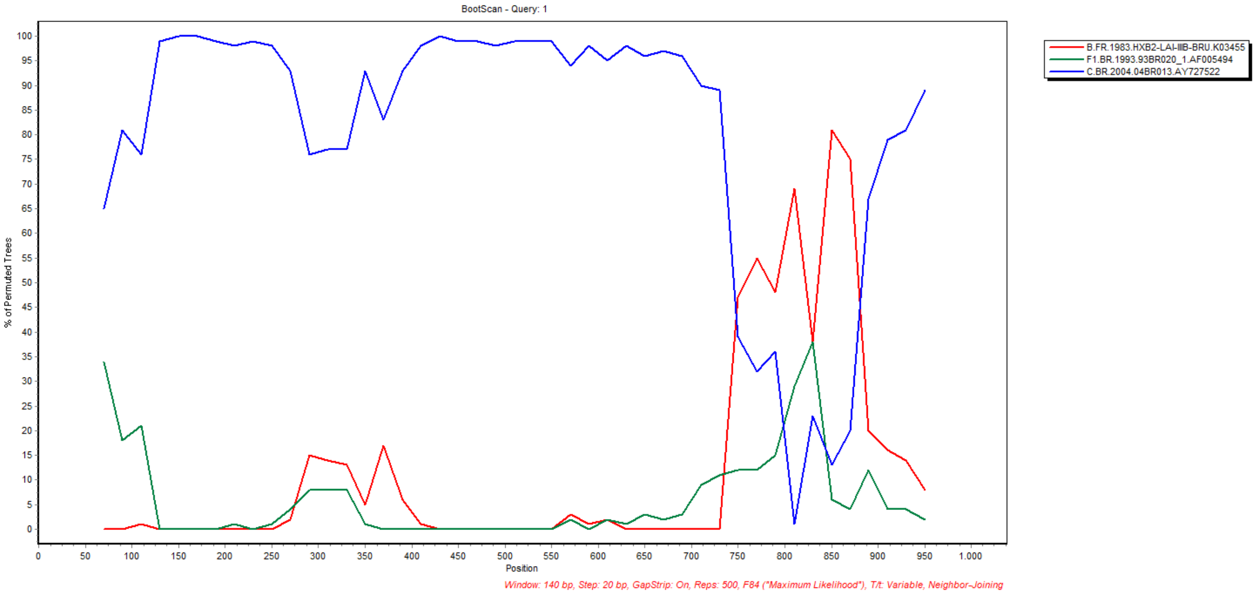

16BRMA04390

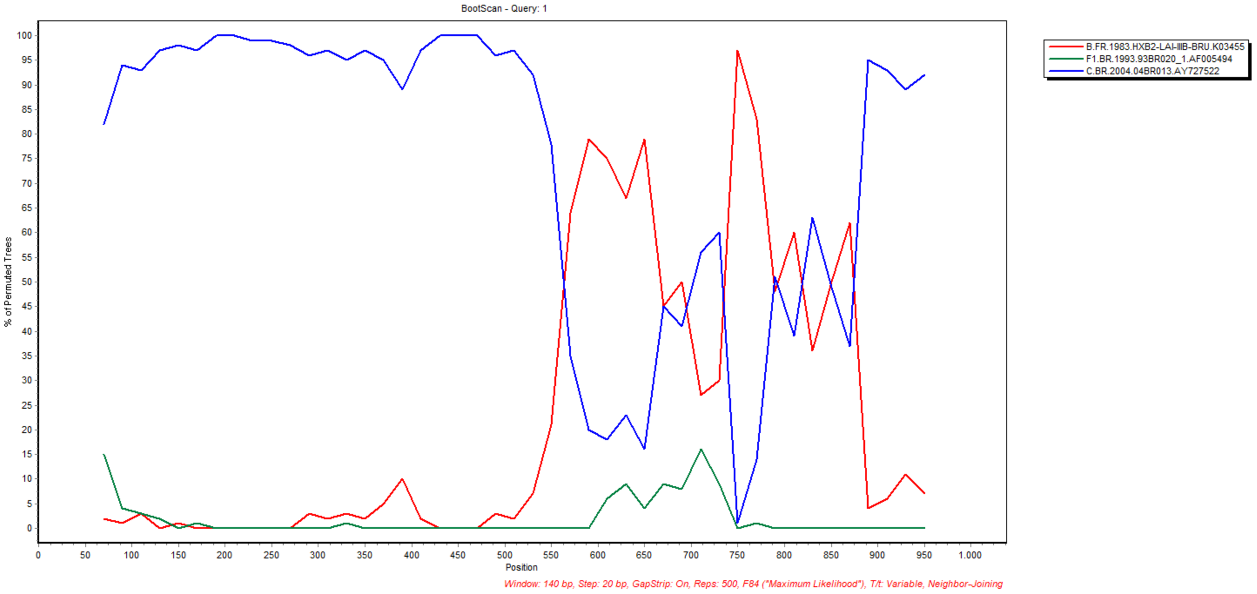


15BRMA0501

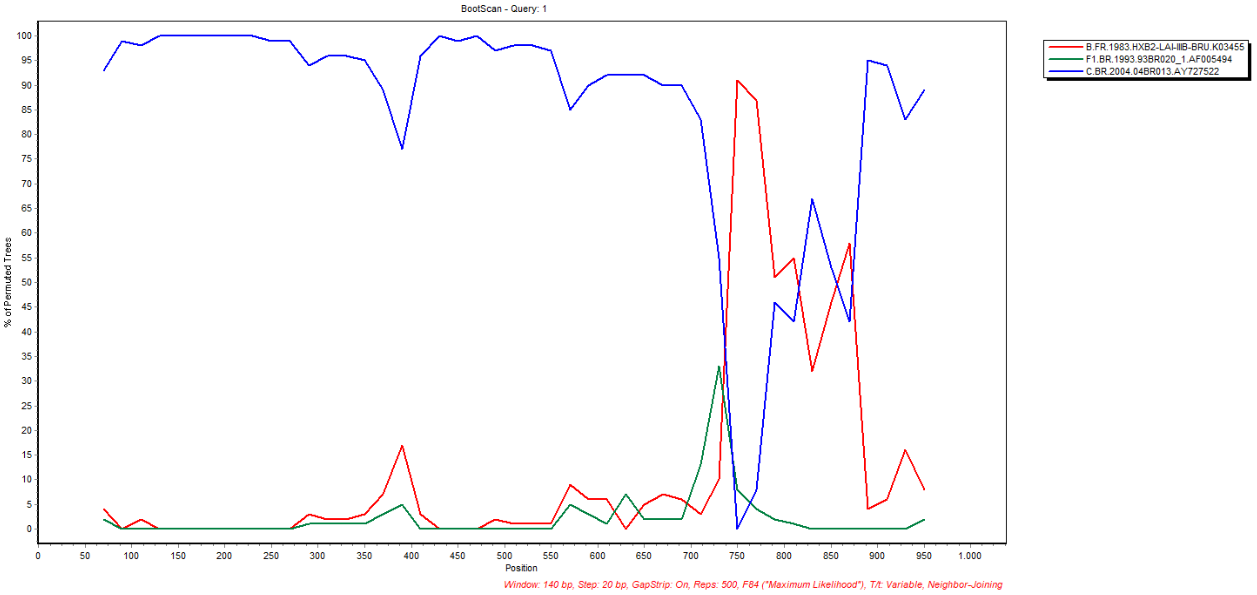


Figure S1 - Patterns of intersubtype genetic recombination of BC recombinants. Recombination analysis performed by bootscanning methodology using SIMPLOT software. Reference strains: B.FR.1983.HXB2-LAI-IIIB-BRU.K03455 (subtype B, red),

F1.BR.1993.93BR020-1. AF005494 (subtype F, green), C.BR.2004.04BR013.AY727522 (subtype C, blue).

## SUPPLEMENTARY MATERIAL S2

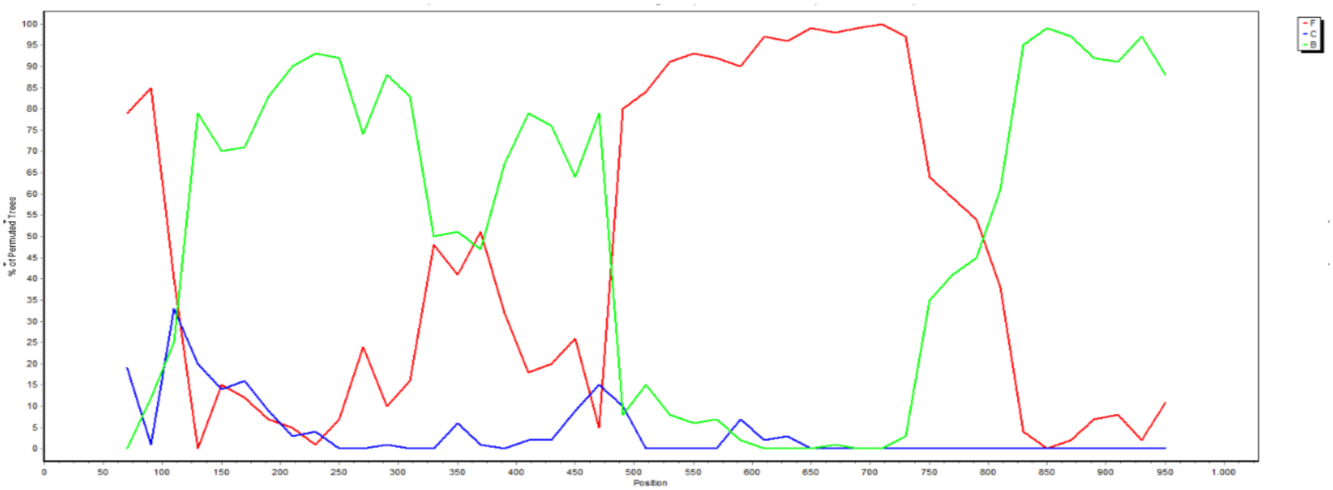


Recombination pattern Cluster 2 (coloured by red in figure 2)

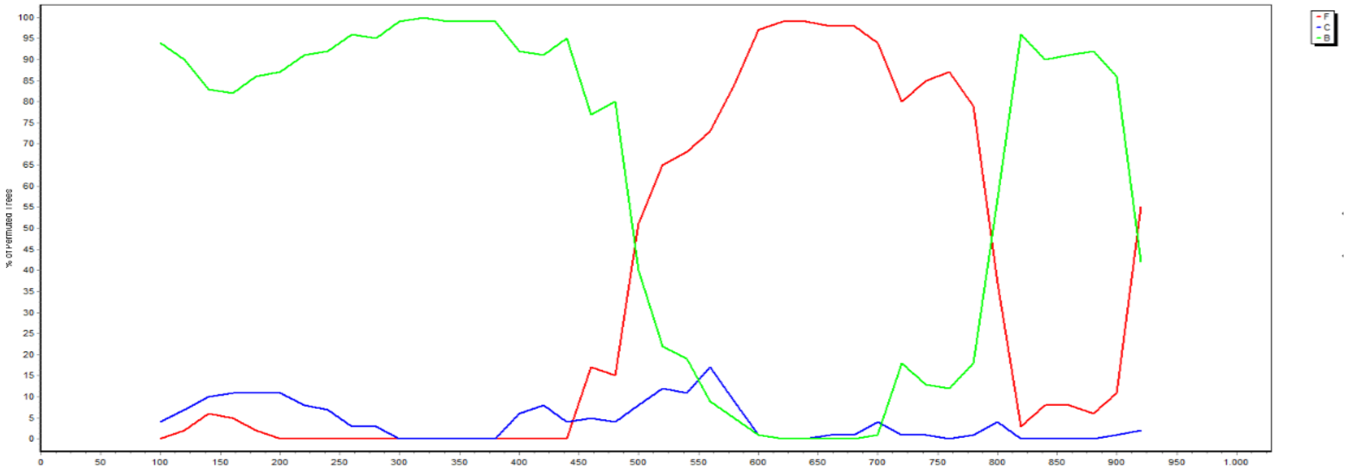


Figure S2 - Patterns of intersubtype genetic recombination of URFs BF presented in two major clusters. Cluster 1 with 14 query sequences and Cluster 2 with 9 one. Recombination analysis performed by bootscanning methodology using SIMPLOT software. Reference strains: B.FR.1983.HXB2-LAI-IIIB-BRU.K03455 (subtype B, red), F1.BR.1993.93BR020-1. AF005494 (subtype F, green), C.BR.2004.04BR013.AY727522 (subtype C, blue).

Recombination pattern Cluster 1 (Coloured by green in figure 2)

## SUPPLEMENTARY MATERIAL Figure S3

16BRMA10040

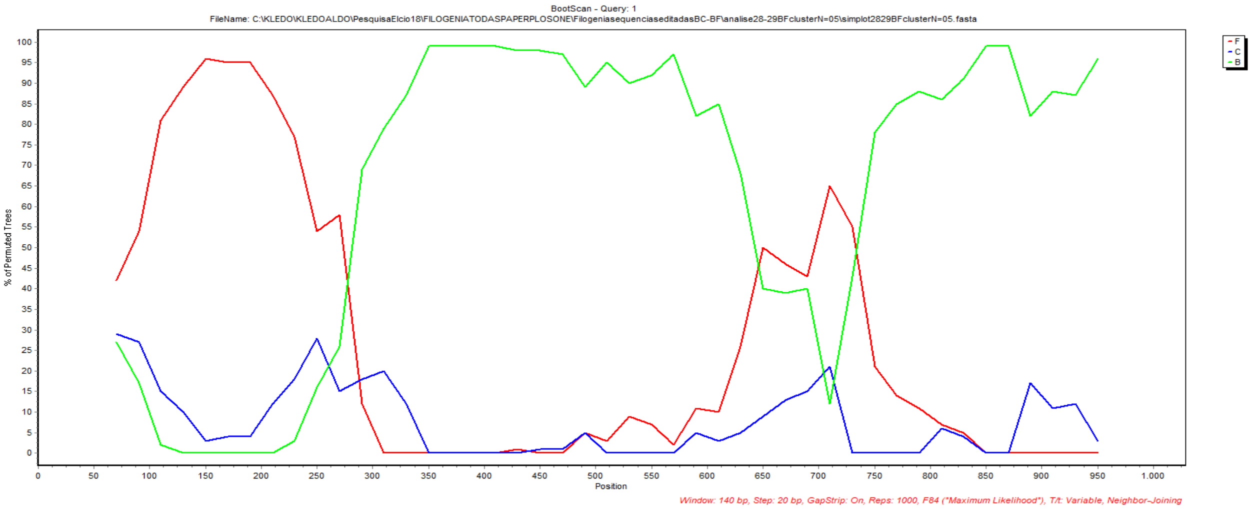


16BRMA09681

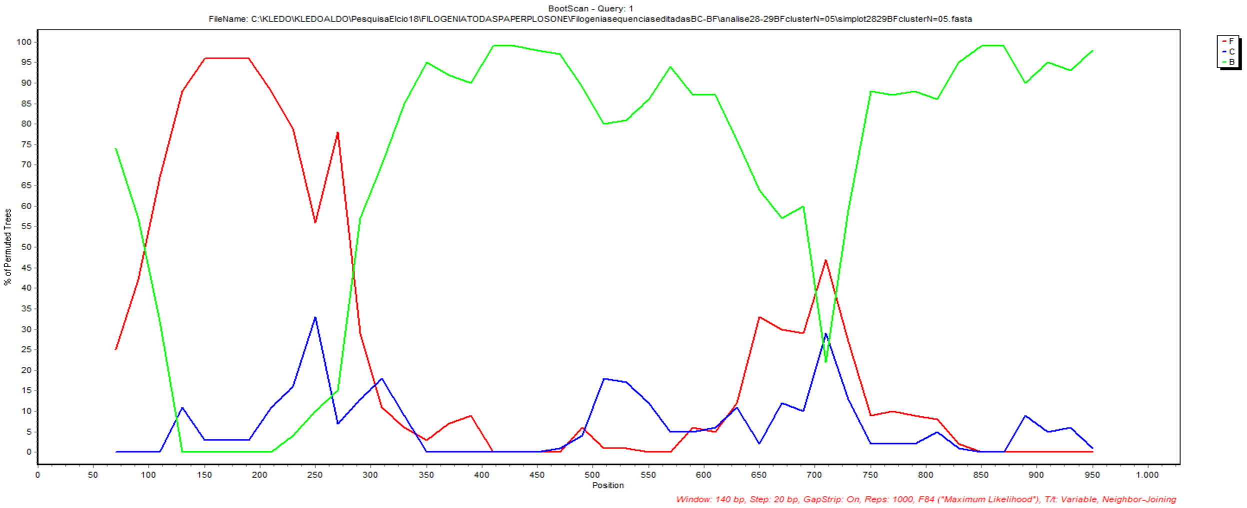


13BRMA0184

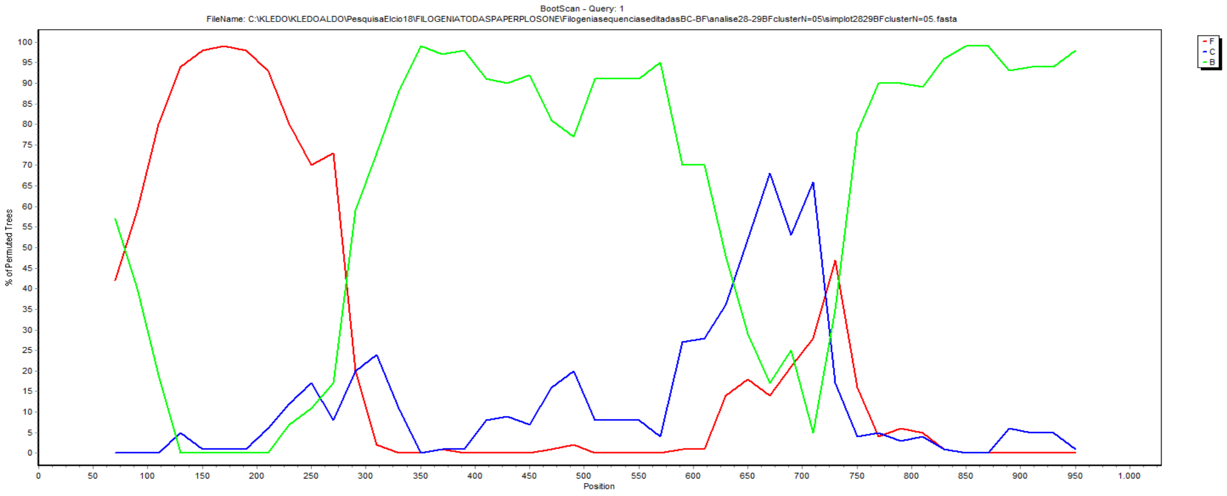


16BRMA06276

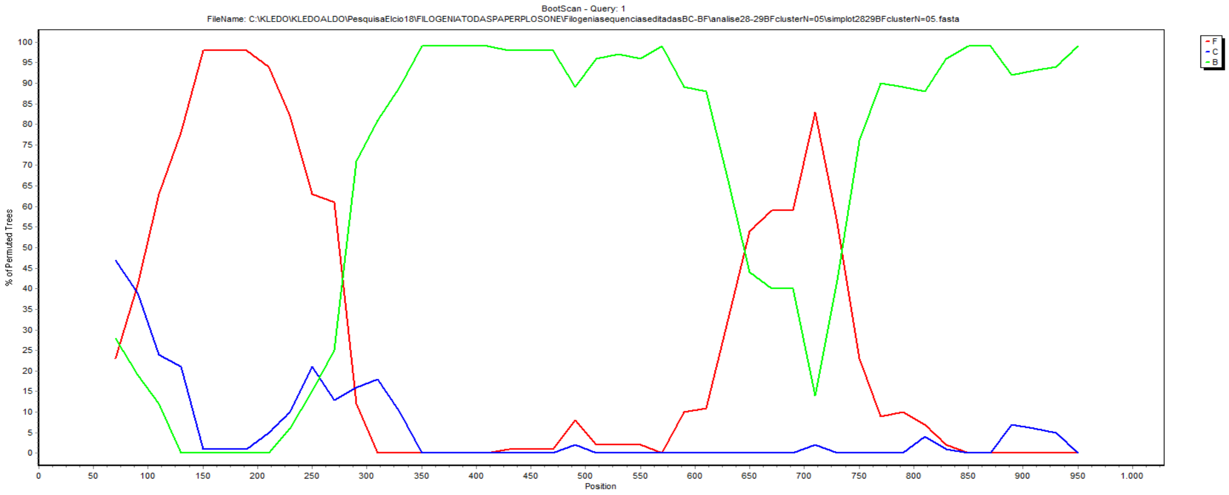


16BRMA06280

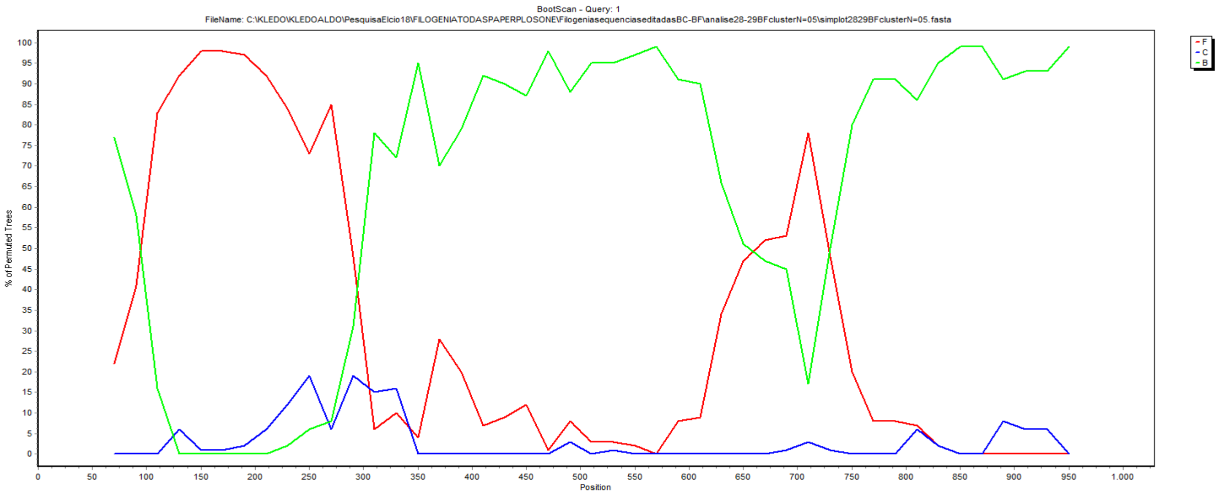

14BRMA0021

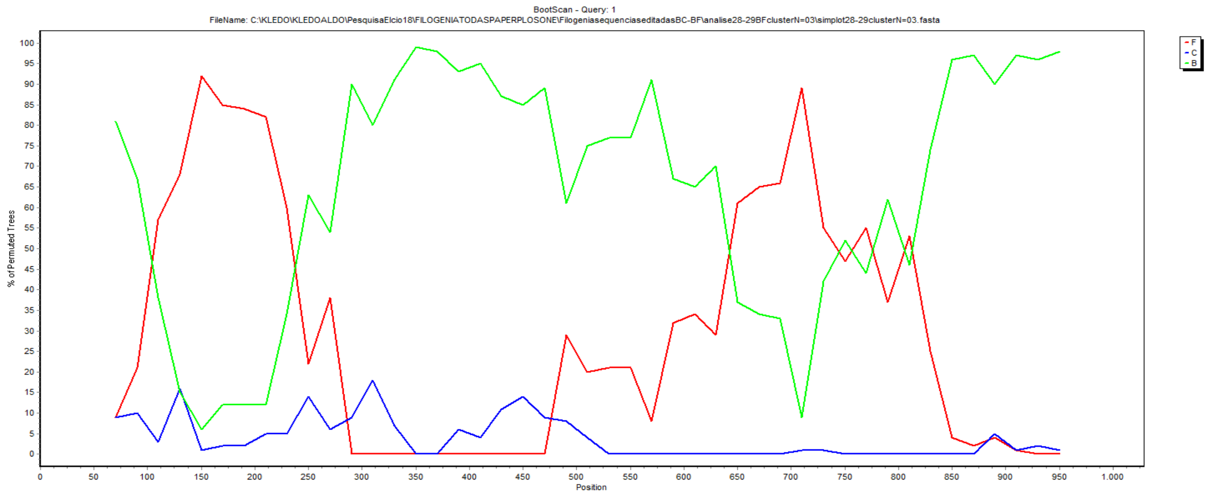


15BRMA0416

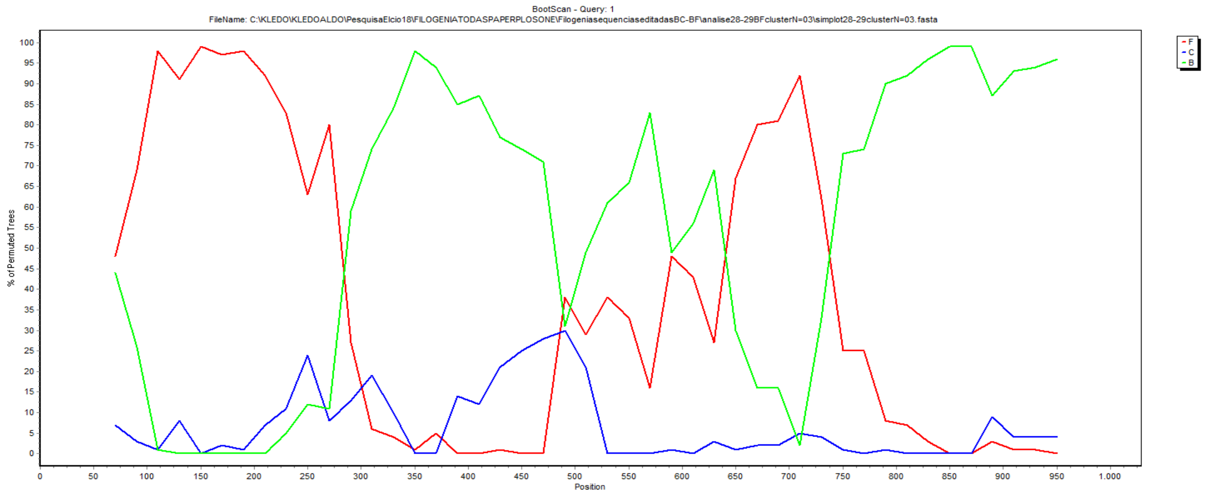


13BRMA0508

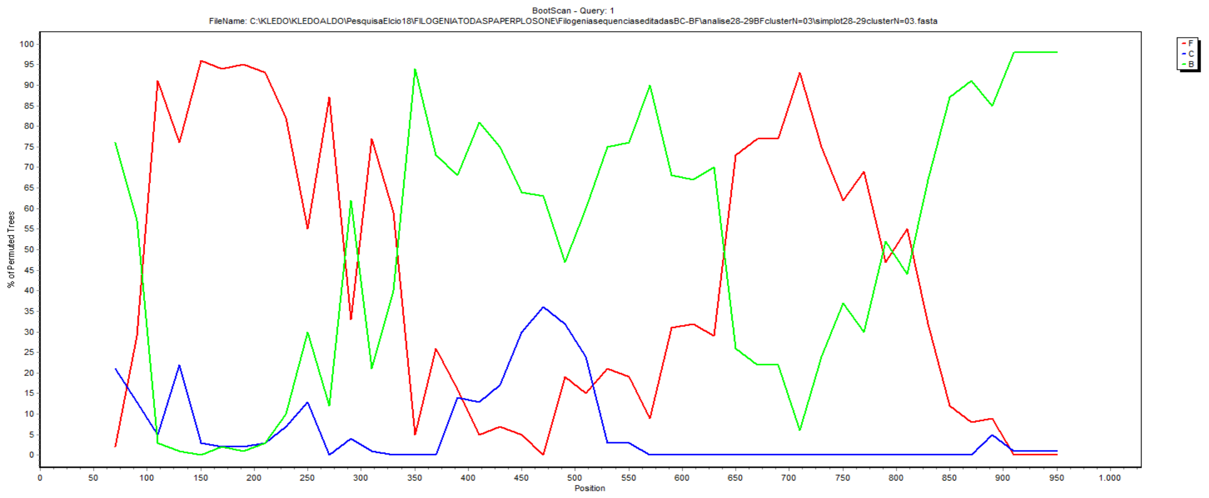

Figure S3 - Patterns of intersubtype genetic recombination of URFs BF recombinants related with CRFs 28 e 29_BF. Recombination analysis performed by bootscanning methodology using SIMPLOT software. Reference strains: B.FR.1983.HXB2-LAI-IIIB-BRU.K03455 (subtype B, red), F1.BR.1993.93BR020-1. AF005494 (subtype F, green), C.BR.2004.04BR013.AY727522 (subtype C, blue).

Cluster C (figure 2):
